# Are comorbidities associated with long-term survival of lung cancer? A population-based cohort study from French cancer registries

**DOI:** 10.1186/s12885-018-5000-7

**Published:** 2018-11-12

**Authors:** A. Seigneurin, P. Delafosse, B. Trétarre, A. S. Woronoff, M. Velten, P. Grosclaude, A. V. Guizard, B. Lapôtre-Ledoux, S. Bara, F. Molinié, M. Colonna

**Affiliations:** 1 Isère Cancer Registry, CHU Grenoble, Grenoble, France; 2 Grenoble Alpes University, Techniques de l’Ingénierie Médicale et de la Complexité – Informatique Mathématiques et Applications Grenoble, Unité Mixte de Recherche 5525, Grenoble, France; 3 Medical evaluation unit, CHU Grenoble Alpes, Grenoble, France; 4 Hérault Cancer Registry, Montpellier, France; 5 Doubs Cancer Registry, CHU Besançon, Besançon, France; 6 Bas-Rhin Cancer Registry, Université de Strasbourg, Strasbourg, France; 7 Tarn Cancer Registry, Institut Claudius Regaud, IUCT-O, Registre des cancer du Tarn, Toulouse, France; 8 LEASP - UMR 1027 Inserm-Université Toulouse III, Toulouse, France; 9 Calvados Cancer Registry, CLCC François Baclesse, Caen, France; 10 Somme Cancer Registry, CHU Amiens, Amiens, France; 11 Manche Cancer Registry, CH du Cotentin, Cherbourg en Cotentin, France; 12 Loire-Atlantique and Vendée Cancer Registry, CHU Nantes, Nantes, France

**Keywords:** Lung cancer, Prognostic factors, Net survival, Histological type, Population-based study

## Abstract

**Background:**

Survival rates of lung cancer remains poor and the impact of comorbidities on the prognosis is discussed. The objective of this study was to assess if the Charlson Comorbidity Index (CCI) was associated with 8-year survival rates by histological type.

**Methods:**

A cohort study was conducted using randomly selected cases from 10 French cancer registries. Net survival rates were computed using the Pohar-Perme estimator of the net cumulative rate. Three Cox models were independently built for adenocarcinomas, squamous cell and small cell cancers to estimate prognostic factors including CCI grade.

**Results:**

A total of 646 adenocarcinomas, 524 squamous cell and 233 small cell cancers were included in the analysis. The net 8-year survival rate ranged from 12.6% (95% CI: 9.8–15.4%) for adenocarcinomas and 13.4% (95% CI: 10.1–16.7%) for squamous cell carcinomas, to 3.7% (95% CI: 1.1–6.3%) for small cell cancers. Observed and net survival rates decreased for CCI grades ≥3 for all histological group considered.

After adjustment for sex, age group, stage and diagnostic mode, CCI grades 1 (HR = 1.6 [95% CI: 1.1–2.3]), 2 (HR = 1.7 [95% CI: 1.1–2.7]) and ≥ 3 (HR = 2.7 [95% CI: 1.7–4.4]) were associated with lower survival rates only for small cell cancers.

**Conclusion:**

After adjustment for age, sex, stage and diagnostic mode, the presence of comorbidity based on CCI grades 1–2 and ≥ 3 was associated with lower survival rates for small cell cancers whereas no differences were observed for adenocarcinomas and squamous cell cancers.

## Background

Lung cancer is the leading cause of cancer and cancer death worldwide with 1.82 million new cases by 2012, representing 12.9% of all new cancers, and 1.6 million deaths (19.5% of the total) [1]. In France, nearly 39,500 lung cancers were diagnosed and 30,000 individuals died from lung cancer in 2012, representing the 4th rank for incidence and the 1st rank for mortality [2]. Data from cancer registries contributing to the International Agency for Research on Cancer (IARC) database showed that lung cancer incidence rates have peaked among men in many areas of the world, whereas rates among women continue to rise [3].

The prognosis of lung cancer remains poor and the improvement in survival that have been realized in other cancers have yet to be achieved in lung cancer. Indeed, the 5-year relative survival rate in US cancer registries for lung cancer diagnosed in 2008–2014 was 18.6% [4]. In France, the 5-year net survival rate for lung cancer diagnosed in 2005–2010 was 17% (95% CI: 16–17%) and the 10-year net survival was only 10% for the diagnoses in the 1999–2004 period [5]. Several prognostic factors for non-small cell cancers have been identified [6, 7]. Stage at diagnosis remains one of the main factor [8] and other potential prognostic factors include performance status [9, 10], increasing age [10, 11], male gender [12] and low socio economic status, [11] whereas the histological sub-type of non-small cell cancer remains controversial [13, 14]. For small cell cancers, disease extent and performance status were identified as independent prognostic factors [15].

The association of comorbidity based on the Charlson Comorbidity Index (CCI) with lung cancer prognosis remains discussed. Compared to an absence of comorbidity, lower survival was found for CCI grades 1–2 and ≥ 3 among non-small cell cancers who underwent curative surgery [10]. An analysis of a Spanish hospital database that included all histological types of lung cancers showed an impaired prognosis only for CCI grades ≥3 [16]. CCI grades 1–2 and ≥ 3 were associated with lower survival rates only for patients diagnosed with a low stage lung cancer (staged as 1 or 2) in a population-based study from the Danish Lung Cancer Registry [17]. On the other hand, Ganti et al. [18] did not find differences in survival for different CCI grades among all histological types of lung cancer treated in a US hospital. Few studies were conducted to estimate the association of CCI with long term survival rates using population-based data, separately for each histological type.

The objective of this study was to assess if CCI was associated with 8-year survival using population-based data, separately for adenocarcinomas, squamous cell carcinomas and small cell cancers.

## Methods

### Study design and setting

A retrospective cohort study was conducted using cases recruited from cancer registries. The original data from 9 cancer registries of the French network of cancer registries (FRANCIM) which covered 10 French administrative entities (Départements of Calvados, Doubs, Hérault, Isère, Loire-Atlantique, Manche, Bas-Rhin, Somme, Tarn, Vendée) were analysed.

### Participants

Participants were randomly selected from the databases of cancer registries on the basis of the day and month of birth of patients.

The total number of cases included in the analysis was computed considering the comparison of survival rates between two groups using the Log rank test. The primary objective was to assess if CCI was associated with 8-year survival among each histological type of lung cancer. Considering 4 groups of CCI grades, a total of 67 cases per group corresponding to 268 cases per histological type was necessary to detect a Hazard Ratio of 0.6 with a 8-year survival rate of 10% in the reference group, a 80% statistical power and a 5% alpha threshold. In a previous study, small cell cancer was the less frequent histological group representing between 12 and 18% of all lung cancers, depending on period of diagnosis and sex [19]. Consequently, the necessary number of lung cancers in the study was 1787 to obtain at least 268 cases of small cell cancers (268/0.15). As a result, the objective was to include 1800 cases corresponding to 200 cases for each cancer registry.

Inclusion criteria were as follows: invasive lung cancers classified as C34 in the International Classification of Disease for Oncology - 3 [20], diagnosed in 2004 among individuals who lived in one of the 10 administrative entities covered by the cancer registries. Only adenocarcinomas, squamous cell cancers and small cell cancers were considered for the analysis since other histological types were less frequent and constituted an heterogeneous group.

### Data collection

Cancer registries routinely collected lung cancer cases from different sources including histopathology laboratories, oncology departments, multidisciplinary meetings, and computerized hospital discharge databases. For this specific study, a supplementary collection of data in medical files was carried out to follow-up participants and to collect diagnostic mode, tobacco smoking, comorbidity, stage at diagnosis and treatment. Specific training sessions were implemented to provide a consistent collection of data among the cancer registries.

Stage for small-cell cancers was defined as limited (cancer remaining in one side of the chest including lung and lymph nodes on the same side of the chest) or extensive (cancer spreading widely throughout the lung, to the other lung, to lymph nodes on the other side of the chest, or to distant organs). Non-small cell cancers were categorized according to the TNM staging system [21] as stage 1 (T1-T2 N0 M0), stage 2 (T1-T2 N1 M0; T3 N0 M0), stage 3 (T1-T2-T3 N2 M0; T3 N1 M0; T1-T2-T3-T4 N3 M0; T4 N0-N1-N2-N3 M0) and stage 4 (T1-T2-T3-T4 N0-N1-N2-N3 M1).

The presence of comorbidities was collected using the Charlson Comorbidity Index (CCI) [22]. Briefly, the CCI is a simple and valid method of classifying comorbidity based on a weighted index that takes into account the number and the seriousness of comorbid condition. We collected data on the presence of comorbid condition at the time of diagnosis from hospitals and GP’s medical files. Performance status was not included considering the important proportion of missing values in medical files.

An active search for the vital status at June 30, 2013 was carried out for all cases included in the study. The information was collected for individuals with a birthplace known by an electronic request to the Répertoire National d’Identification des Personnes Physiques which collects data on deaths in France. In case of missing birthplace, other sources of information on the vital status were used such as medical records.

### Study outcomes

The main study endpoint was all cause of death during a 8-year follow-up period.

### Statistical analyses

Observed and net survival rates, i.e. survival rates that would have been observed if lung cancer was the only cause of death in the population, were computed by histological type and CCI grades. Observed survival rates were obtained using the Kaplan-Meier method. Considering the unavailability and unreliability of causes of deaths, net survival rates were computed using the Pohar-Perme estimator of the net cumulative rate which did not require knowing causes of deaths [23]. Indeed, lung cancer mortality was deduced from the all-cause mortality of the study group and the “expected” mortality of a disease-free group. This expected mortality was assumed to reflect correctly the mortality due to other causes than lung cancer and was obtained from the general population life tables [24].

Three Cox models were independently built for adenocarcinomas, squamous cell cancers and small cell cancers to estimate the adjusted hazard ratio of death for different CCI grades. The proportional-hazards assumption was assessed graphically and tested on the basis of Schoenfeld residuals. Time-varying variables were introduced if the proportional hazard assumption was not met to estimate different effects during the 0–1 year and 1–8 years period after diagnosis. For each histological type, a full model was considered including sex, age group, CCI, stage, and diagnostic mode since asymptomatic cancers could be associated with better survival rates due to lead time, length bias and overdiagnosis.

## Results

A total of 1751 lung cancer cases diagnosed in 2004 were collected. Adenocarcinoma was the most frequent histological type (36.9%), followed by squamous cell carcinoma (29.9%) and small cell carcinomas (13.3%). Other histological types represented 15.6% of cases and 4.2% had no cytological or histological diagnosis. Observed and net 8-year survival rates for all histological groups and stages combined were 9.7% (95% CI: 8.4–11.2%) and 11.2% (95% CI: 9.5–12.8%), respectively.

The present study focused on adenocarcinomas, squamous cell and small cell cancers. The main characteristics of the participants are summarized in Table 1. The repartition of gender differed by histological type: the proportion of men was 68.1% for adenocarcinomas, 90.5% for squamous cell cancers and 82.0% for small cell cancers. For each histological type, the upper lobe was the most frequent topography and pulmonary symptom was the most frequent diagnostic mode. The most frequent comorbid conditions were similar for the three histological types of lung cancers considered with chronic obstructive pulmonary disease, followed by peripheral vascular disease, and congestive heart failure. By June 30, 2013, less than 1.5% of individuals were lost to follow-up (Table 2).

**Table 1:** Characteristics of the 1403 individuals diagnosed with lung cancers in 2004 categorized as adenocarcinomas, squamous cell carcinomas and small cell cancers and recruited among 10 French cancer registries

	Adenocarcinomas	Squamous cell carcinomas	Small cell carcinomas
	*N* = 646 (100.0%)	*N* = 524 (100.0%)	*N* = 233 (100.0%)
Sex			
Male	440 (68.1%)	474 (90.5%)	191 (82.0%)
Female	206 (31.9%)	50 (9.5%)	42 (18.0%)
Age group			
< 50	89 (13.8%)	21 (4.0%)	19 (8.1%)
50–59	186 (28.8%)	118 (22.5%)	68 (29.2%)
60–69	162 (25.1%)	142 (27.1%)	57 (24.5%)
70–79	163 (25.2%)	173 (33.0%)	70 (30.0%)
≥ 80	46 (7.1%)	70 (13.4%)	19 (8.1%)
Comorbid conditions^a^			
Chronic obstructive pulmonary disease	130 (20.1%)	161 (30.7%)	54 (23.2%)
Peripheral vascular disease	86 (13.3%)	77 (14.7%)	28 (12.0%)
Congestive heart failure	54 (8.4%)	75 (14.3%)	23 (9.9%)
Charlson Comorbidity Index			
0	319 (49.4%)	195 (37.2%)	107 (45.9%)
1	159 (24.6%)	157 (30.0%)	61 (26.2%)
2	84 (13.0%)	79 (15.1%)	31 (13.3%)
≥ 3	62 (9.6%)	75 (14.3%)	24 (10.3%)
Unknown	22 (3.4%)	18 (3.4%)	10 (4.3%)
Topography			
Main bronchus	22 (3.4%)	59 (11.3%)	24 (10.3%)
Upper lobe	341 (52.8%)	254 (48.5%)	109 (46.8%)
Middle lobe	26 (4.0%)	14 (2.7%)	12 (5.1%)
Lower lobe	153 (23.7%)	133 (25.4%)	43 (18.5%)
Overlapping lesion	33 (5.1%)	25 (4.8%)	26 (11.2%)
Not specified	71 (11.0%)	39 (7.4%)	19 (8.1%)
Stage			
Non-small cell – Surgery			
1	75 (41.0%)	52 (37.4%)	–
2	18 (9.8%)	39 (28.1%)	–
3	62 (33.9%)	45 (32.4%)	–
4	26 (14.2%)	2 (1.4%)	–
Unknown	2 (1.1%)	1 (0.7%)	–
Non-small cell – No surgery			
1	8 (1.7%)	16 (4.2%)	–
2	2 (0.4%)	5 (1.3%)	–
3	87 (18.8%)	138 (36.0%)	–
4	354 (76.5%)	198 (51.7%)	–
Unknown	12 (2.6%)	26 (6.8%)	–
Small cell – Surgery			
Limited	–	–	6 (85.7%)
Extensive	–	–	1 (14.3%)
Unknown	–	–	0 (0.0%)
Small cell – No surgery			
Limited	–	–	61 (27.1%)
Extensive	–	–	155 (68.9%)
Unknown	–	–	9 (4.0%)
Diagnostic mode			
Incidental diagnosis	76 (11.8%)	57 (10.9%)	19 (8.1%)
Pulmonary symptoms	360 (55.7%)	350 (66.8%)	143 (61.4%)
Other symptoms	169 (26.2%)	96 (18.3%)	63 (27.0%)
Surveillance of high-risk patients	29 (4.5%)	12 (2.3%)	2 (0.9%)
Unknown	12 (1.9%)	9 (1.7%)	6 (2.6%)
Tobacco smoking			
Current smoker	274 (42.4%)	224 (42.7%)	123 (52.8%)
Former-smoker	227 (35.1%)	248 (47.3%)	89 (38.2%)
Never smoker	85 (13.2%)	15 (2.9%)	1 (0.4%)
Unknown	60 (9.3%)	37 (7.1%)	20 (8.6%)
Absence of treatment			
Yes	78 (12.1%)	84 (16.0%)	34 (14.6%)
Unknown	0 (0.0%)	0 (0.0%)	0 (0.0%)

^a^The three most frequent comorbid diseases were reported

**Table 2:** Observed and net survival rates (% (95% CI)) by histological types among lung cancers diagnosed in 2004 and recruited in 10 French cancer registries

	Adenocarcinoma *N* = 646	Squamous cell carcinoma *N* = 524	Small cell carcinoma *N* = 233
Number of person-years	1374.5	1020.7	264.8
Percentage of dead within 8 years	88.5%	88.7%	96.6%
Lost to follow-up at 8 years	1.4%	1.3%	0.4%
1-year survival rate			
Observed	49.4% (45.5–53.2%)	43.3% (39.0–47.5%)	31.8% (25.9–37.8%)
Net	50.1% (46.2–54.0%)	44.5% (40.1–48.8%)	32.2% (26.2–38.3%)
5-year survival rate			
Observed	15.8% (13.1–18.8%)	14.9% (12.0–18.1%)	3.9% (1.9–6.9%)
Net	17.1% (14.1–20.2%)	16.5% (13.1–19.9%)	4.1% (1.6–6.7%)
8-year survival rate			
Observed	11.2% (8.9–13.8%)	11.2% (8.7–14.1%)	3.4% (1.6–6.4%)
Net	12.6% (9.8–15.4%)	13.4% (10.1–16.7%)	3.7% (1.1–6.3%)

Net 8-year survival rates, reported in Table 2, remained low for each histological group: 12.6% (95% CI: 9.8–15.4%) for adenocarcinomas, 13.4% (95% CI: 10.1–16.7%) for squamous cell cancers and 3.7% (95% CI: 1.1–6.3%) for small cell cancers. Observed and net survival rates were close for all histological types.

Observed and net survival rates decreased when the CCI grade was ≥3 for all histological group (Table 3). Univariate and multivariate prognostic factors obtained from Cox models are reported in Table 4. After adjustment for sex, age group, stage and diagnostic mode, CCI grades 1, 2 and ≥ 3 were associated with lower survival rates only for small cell cancers, whereas no differences were observed for adenocarcinomas and squamous cell cancers. Stage at diagnosis remains the main prognostic factor for all histological types. Females were associated with higher survival rates only for adenocarcinomas and age at diagnosis ≥80 was associated with lower survival rates for the 3 histological types. We did not include variables related to treatments in our model due to a high level of collinearity with stage. The proportion of patients who did not receive any curative treatments for CCI grades 0, 1, 2 and ≥ 3 were 10.3, 13.1, 16.1 and 29.2% for small cell cancers, 11.9, 11.9, 11.9 and 16.1% for adenocarcinomas, 12.3, 15.3, 20.2 and 21.3% for squamous cell cancers, respectively.

**Table 3:** Observed and net 8-year survival rates (% (95% CI)) by histological type and Charlson Comorbidity Index (CCI) grade for adenocarcinomas, squamous cell carcinomas and small cell cancers diagnosed in 2004 and recruited among 10 French cancer registries

CCI grade	Adenocarcinomas (*n* = 646)		Squamous cell cancers (*n* = 524)		Small cell cancers (*n* = 233)	
	Observed survival	Net survival	Observed survival	Net survival	Observed survival	Net survival
0	12.3% (9.0–16.2%)	13.7% (9.7–17.8%)	12.3% (8.2–17.3%)	14.1% (8.7–19.4%)	3.7% (1.2–8.6%)	4.1% (0.2–8.0%)
1	13.2% (8.5–19.0%)	15.1% (9.1–22.2%)	10.2% (6.1–15.5%)	12.6% (6.8–18.4%)	3.3% (0.6–10.1%)	3.5% (0.0–7.8%)
2	11.9% (6.1–19.8%)	14.0% (5.7–22.2%)	13.7% (7.2–22.3%)	17.4% (7.9–26.8%)	3.2% (0.2–14.1%)	3.3% (0.0–8.4%)
≥ 3	1.6% (0.1–7.6%)	1.7% (0.0–4.5%)	8.0% (3.3–15.5%)	10.1% (2.3–17.9%)	0.0% (0.0–0.0%)	0.0% (0.0–1.0%)

**Table 4:** Factors obtained from a cox model and associated with observed mortality for adenocarcinomas, squamous cell cancers and small cell cancers diagnosed in 2004 and recruited among 10 French cancer registries

	Adenocarcinomas (n = 646)				Squamous cell cancers (n = 524)				Small cell cancers (n = 233)			
	Time (years)^**a**^	Unadjusted Hazard Ratios	Time (years) ^**a**^	Adjusted Hazard Ratios	Time (years) ^**a**^	Unadjusted Hazard Ratios	Time (years) ^**a**^	Adjusted Hazard Ratios	Time (years) ^**a**^	Unadjusted Hazard Ratios	Time (years) ^**a**^	Adjusted Hazard Ratios
Sex												
Male		1		1		1		1		1		1
Female	0–8	0.8 (0.6–0.9)	0–8	0.7 (0.6–0.8)	0–8	1.1 (0.8–1.6)	0–8	1.3 (1.0–1.8)	0–8	0.8 (0.6–1.1)	0–8	0.7 (0.5–1.0)
Age group												
< 50		1		1		1		1		1		1
50–59	0–8	1.1 (0.9–1.5)	0–8	1.0 (0.7–1.3)	0–8	1.0 (0.6–1.6)	0–8	0.9 (0.6–1.5)	0–8	1.0 (0.6–1.8)	0–8	0.9 (0.5–1.6)
60–69	0–8	1.3 (1.0–1.7)	0–8	1.1 (0.8–1.4)	0–8	0.9 (0.5–1.4)	0–8	0.9 (0.5–1.5)	0–8	1.4 (0.8–2.4)	0–8	1.0 (0.6–1.7)
70–79	0–8	1.2 (0.9–1.6)	0–8	1.2 (0.9–1.6)	0–8	1.1 (0.7–1.8)	0–8	1.4 (0.8–2.3)	0–8	1.9 (1.1–3.2)	0–8	1.3 (0.8–2.3)
≥ 80	0–8	2.2 (1.5–3.2)	0–8	2.9 (2.0–4.3)	0–8	1.9 (1.1–3.2)	0–8	1.9 (1.1–3.2)	0–8	3.1 (1.6–6.1)	0–8	2.5 (1.3–4.9)
Charlson Comorbidity Index												
0		1		1		1		1		1		1
1	0–8	1.0 (0.8–1.2)	0–8	1.0 (0.8–1.3)	0–8	1.0 (0.8–1.3)	0–8	1.0 (0.8–1.3)	0–8	1.3 (0.9–1.8)	0–8	1.6 (1.1–2.3)
2	0–8	0.9 (0.7–1.2)	0–8	0.8 (0.6–1.1)	0–8	0.9 (0.7–1.2)	0–8	0.9 (0.6–1.2)	0–8	1.4 (0.9–2.1)	0–8	1.7 (1.1–2.7)
≥ 3	0–8	1.3 (1.0–1.7)	0–8	1.2 (0.9–1.6)	0–8	1.1 (0.8–1.4)	0–8	0.9 (0.6–1.2)	0–8	2.4 (1.5–3.7)	0–8	2.7 (1.7–4.4)
Unknown	0–8	2.1 (1.4–3.3)	0–8	1.6 (1.0–2.6)	0–8	1.1 (0.7–1.9)	0–8	0.9 (0.6–1.6)	0–8	1.4 (0.7–2.8)	0–8	1.2 (0.6–2.5)
Stage (non-small cell cancers)												
1		1		1		1		1		–		–
2	0–8	1.1 (0.6–2.2)	0–1	0.4 (0.1–3.3)	0–8	1.1 (0.7–1.7)	0–8	1.1 (0.7–1.8)		–		–
			1–8	1.3 (0.0–90.3)								
3	0–8	3.2 (2.2–4.5)	0–1	3.3 (1.6–6.7)	0–8	2.6 (1.8–3.7)	0–8	2.8 (2.0–4.0)		–		–
			1–8	3.3 (0.7–15.3)								
4	0–8	6.9 (5.0–9.6)	0–1	8.7 (4.4–17.1)	0–8	6.1 (4.3–8.7)	0–8	6.7 (4.6–9.6)		–		–
			1–8	6.4 (1.5–27.5)								
Unknown	0–8	5.6 (3.0–10.5)	0–1	8.2 (3.2–21.0)	0–8	7.0 (4.3–11.4)	0–8	7.2 (4.4–11.7)		–		–
			1–8	3.2 (0.3–33.2)								
Stage (small cell cancers)												
Limited		–		–		–		–		1		1
Extensive		–		–		–		–	0–8	2.7 (1.9–3.6)	0–8	3.1 (2.2–4.4)
Unknown		–		–		–		–	0–8	6.2 (3.1–12.5)	0–8	4.7 (2.1–10.2)
Diagnostic mode												
Pulmonary symptoms		1		1		1		1		1		1
Incidental diagnosis	0–1	0.8 (0.6–1.0)	0–1	0.7 (0.5–1.1)	0–8	0.9 (0.7–1.3)	0–8	0.9 (0.7–1.2)	0–8	0.7 (0.4–1.1)	0–8	0.8 (0.4–1.3)
	1–8	0.7 (0.4–1.3)	1–8	1.0 (0.4–2.5)								
Non-pulmonary symptoms	0–1	1.3 (1.1–1.6)	0–1	1.1 (0.8–1.4)	0–8	1.8 (1.4–2.3)	0–8	1.5 (1.2–1.9)	0–8	1.2 (0.9–1.7)	0–8	1.1 (0.8–1.5)
	1–8	1.6 (0.9–2.6)	1–8	0.9 (0.5–1.7)								
Surveillance of high-risk patients	0–1	0.5 (0.3–0.7)	0–1	0.4 (0.2–1.1)	0–8	1.0 (0.6–1.8)	0–8	1.1 (0.6–2.0)	0–8	1.0 (0.2–4.1)	0–8	2.0 (0.5–8.7)
	1–8	0.3 (0.1–0.8)	1–8	1.2 (0.1–10.4)								
Unknown	0–1	1.4 (0.8–2.5)	0–1	1.0 (0.5–2.4)	0–8	1.3 (0.6–2.5)	0–8	1.6 (0.8–3.2)	0–8	2.3 (1.0–5.3)	0–8	1.6 (0.6–4.3)
	1–8	3.4 (0.8–13.7)	1–8	1.9 (0.3–14.2)								

^a^Period of time since diagnosis considered to compute hazard ratios

## Discussion

Eight-year net survival rates remained low for all histological types of lung cancers and CCI grades, ranging from 0.0% (95% CI: 0.0–1.0%) for individuals with a CCI grade ≥ 3 diagnosed with a small-cell carcinoma, to 14.1% (95% CI: 8.7–19.4%) for individuals with a CCI grade 0 diagnosed with a squamous cell carcinoma. We found lower survival rates for individuals with CCI grades 1, 2 and ≥ 3 compared to individuals without comorbidity only for small cell cancers, after adjustment for sex, age group, stage and diagnostic mode.

The main patient and cancer characteristics by histological types are consistent with data from literature, including the high proportion of men among squamous cell lung cancers [25, 26]. Since squamous cell cancer is one of the histological types the most closely associated with smoking, the higher smoking rate among men could explain this finding.

Several hypotheses can be considered to explain the lower survival rates observed for patients with comorbidities. First, the presence of comorbidities could increase the delay of diagnosis of lung cancer implying a worst prognosis. We partly took into account the delay of diagnosis by including stage at diagnosis and diagnostic mode in our analyses but a residual effect could still be observed. Second, the use of sub optimal treatments when CCI grades increased could result in an increase of lung cancer-related deaths. For small cell cancers, the decrease of net survival rates from 3.3% for patients with CCI grade 2 to 0.0% for CCI grade ≥ 3 is consistent with this explanation, as well as the increase with CCI grades of the proportion of patients receiving no curative treatments. Less extensive treatments due to the presence of comorbid conditions could also be an explanation for the impaired survival rates. However, the precise type of treatment was not available in our database to verify this hypothesis. Søgaard et al. in a literature review found that patients with comorbidities were less likely to receive guideline-recommended treatments [27]. Third, lung cancer and its treatment could imply a sub optimal treatment of an active comorbidity as well as an unfavorable evolution of the comorbid condition, resulting in an increase of comorbidity-related deaths. Finally, differences in survival rates by CCI grade could reflect differences in socio-economic status [27] but this information was not available at an individual level in our study.

We found an association between the CCI grade at diagnosis and 8-year survival rates only for small cell cancers. The reason for this finding remains unclear. An increased delay of diagnosis, sub optimal treatments for the cancer and/or the comorbid condition and differences in socio-economic status could be observed more frequently for small cell cancers. Moreover, the effect on mortality of these factors might be more important for small cell lung cancer due to specificities in the natural history, the prognosis and the types of treatment performed compared to non-small cell cancers. In the literature, several authors also found lower survival rates for increasing CCI grades for adenocarcinomas and squamous cell cancers when the analysis was restricted to sub groups of patients. Birim et al. [10] studied resected non-small cell lung cancers performed in an hospital and found that after adjusting for several confounders including age, sex, stage and individual comorbid conditions, survival was lower among patients with CCI grades 1–2 and ≥ 3 compared to patients with no comorbidity. A Danish population-based study found that CCI grade ≥ 3 was associated with lower survival among resected non-small lung cell cancers [28]. Few articles studied the effect of CCI grades on survival rates for small cell cancers. Dalton et al. [17] found that CCI grades 1–2 and ≥ 3 were associated with impaired survival only among lung cancers categorized as stage 1 and 2 in an analysis that included both small cell and non-small cell cancers.

Many studies reported prognostic factors of lung cancer [7, 29, 30]. Several studies reported an effect of sex on prognosis whereas we identified a better prognosis for women only among adenocarcinomas. The Surveillance, Epidemiology, and End Results database from 1975 to 1999 showed lower survival for men based on separate analyses by stage at diagnosis and after adjustment for histological type, treatment and age [12]. For small cell cancers, individual data pooled from first-line treatment trials showed a lower survival for men after adjustment for confounding factors [29]. On the other hand, no sex difference was found for resected non-small cell cancers diagnosed in the Medical University of Innsbruck, Austria [31]. Consistent with our results, several studies found lower survival rates for older patients [12, 29, 32].

The limits of our study should be acknowledged. First, the statistical power might have been too low to identify small effects on survival. Second, we could not exclude misclassification of the CCI related to differences in recording comorbidities in medical files [22]. Moreover, we considered comorbidities at the time of diagnosis in our analyses and we did not take into account the diagnosis of comorbid condition during the follow-up. The frequency of comorbidities increases with age, especially among the age group studied, and this increase of comorbidities could affect mortality. Third, we could not collect the cause of death and the multivariate analysis was based on all-cause mortality. As a result, we could not distinguish the effect of comorbidity on lung cancer deaths and comorbidity-related deaths. Fourth, performance status was not introduced as a prognostic factor in our model since this information was not fully available in medical files. Finally, staging procedures have changed since 2004 including the widespread use of PET scan. However, the main objective was to assess the impact of comorbidities on survival adjusted for other prognosis factors. Stage was only used for adjustment purposes and the estimation of its precise effect on survival was not our main objective.

Our study present several strengths. First, we used population-based data from 10 cancer registries resulting in the absence of selection bias encountered in studies based on data obtained in specialized centres or randomized trials. Differences of cancer characteristics and practice patterns may exist among centres but the study reflects diagnosis of lung cancers and treatments in the general population. Second, we estimated the association of CCI grade and survival considering a long follow-up of 8 years. Third, the proportion of individuals lost to follow-up was low. Finally, we analysed the effect of CCI grades on survival separately for adenocarcinomas, squamous cell cancers and small cell cancers.

## Conclusion

After adjustment for age, sex, stage and diagnostic mode, the presence of comorbidity based on CCI grades 1–2 and ≥ 3 was associated with lower 8-year survival rates for small cell cancers whereas no differences were found for adenocarcinomas and squamous cell cancers. Further studies should be conducted to assess differences of survival by CCI grades among sub groups of adenocarcinomas and squamous cell cancers and to elucidate the mechanisms explaining the lower survival associated with comorbid conditions among small cell lung cancers.
